# Feasibility and predictive performance of the Hendrich Fall Risk Model II in a rehabilitation department: a prospective study

**DOI:** 10.1186/s12913-017-2815-x

**Published:** 2018-01-11

**Authors:** Isabella Campanini, Stefano Mastrangelo, Annalisa Bargellini, Agnese Bassoli, Gabriele Bosi, Francesco Lombardi, Stefano Tolomelli, Mirco Lusuardi, Andrea Merlo

**Affiliations:** 1 Department of Rehabilitation, LAM-Motion Analysis Laboratory, AUSL of Reggio Emilia, S. Sebastiano Hospital, Via Mandriolo Superiore 11, 42015 Correggio, RE Italy; 2 Clinical and Experimental Medicine PhD Program, University of Modena and Reggio Emilia, Modena, Italy; 3 Clinical Governance Unit, AUSL of Reggio Emilia, Correggio, Italy; 4 Department of Biomedical, Metabolic and Neural Sciences, University of Modena and Reggio Emilia, Modena, Italy; 5 Neurological Rehabilitation Unit, AUSL of Reggio Emilia, S. Sebastiano Hospital, Correggio, Italy; 6 Orthopaedic Rehabilitation Unit, AUSL of Reggio Emilia, S. Sebastiano Hospital, Correggio, Italy; 7 Respiratory Rehabilitation Unit, AUSL of Reggio Emilia, S. Sebastiano Hospital, Correggio, Italy

**Keywords:** Fall risk assessment, Henrdich II fall risk model, Inpatients, Rehabilitation, Sensitivity and specificity

## Abstract

**Background:**

Falls are a common adverse event in both elderly inpatients and patients admitted to rehabilitation units. The Hendrich Fall Risk Model II (HIIFRM) has been already tested in all hospital wards with high fall rates, with the exception of the rehabilitation setting. This study’s aim is to address the feasibility and predictive performances of HIIFRM in a hospital rehabilitation department.

**Methods:**

A 6 months prospective study in a Italian rehabilitation department with patients from orthopaedic, pulmonary, and neurological rehabilitation wards. All admitted patients were enrolled and assessed within 24 h of admission by means of the HIIFRM. The occurrence of falls was checked and recorded daily. HIIFRM feasibility was assessed as the percentage of successful administrations at admission. HIIFRM predictive performance was determined in terms of area under the Receiver Operating Characteristic (ROC) curve (AUC), best cutoff, sensitivity, specificity, positive and negative predictive values, along with their asymptotic 95% confidence intervals (95% CI).

**Results:**

One hundred ninety-one patents were admitted. HIIFRM was feasible in 147 cases (77%), 11 of which suffered a fall (7.5%). Failures in administration were mainly due to bedridden patients (e.g. minimally conscious state, vegetative state). AUC was 0.779(0.685–0.873). The original HIIFRM cutoff of 5 led to a sensitivity of 100% with a mere specificity of 49%(40–57%), thus suggesting using higher cutoffs. Moreover, the median score for non-fallers at rehabilitation units was higher than that reported in literature for geriatric non fallers. The best trade-off between sensitivity and specificity was obtained by using a cutoff of 8. This lead to sensitivity = 73%(46-99%), specificity = 72%(65-80%), positive predictive value = 17% and negative predictive value = 97%. These results support the use of the HIIFRM as a predictive tool.

**Conclusions:**

The HIIFRM showed satisfactory feasibility and predictive performances in rehabilitation wards. Based on both available literature and these results, the prediction of falls among all hospital wards, with high risk of falling, could be achieved by means of a unique tool and two different cutoffs: a standard cutoff of 5 in geriatric wards and an adjusted higher cutoff in rehabilitation units, with predictive performances similar to those of the best-preforming pathology specific tools for fall-risk assessment.

## Background

Accidental falls are the major cause of hospital injuries, resulting in extended length of stay and a decline in quality of life [1]. The reported occurrences of falls in acute care settings range from 1,4% in a general hospital to 1,9% for a specialty hospital without paediatric and obstetrical services [2]. Higher rates are reported in neurological, geriatric and rehabilitative wards. In particular, fall occurrences rise from 12,5% in general inpatient rehabilitation settings to 20–30% in general geriatric rehabilitation units, up to 39% in geriatric stroke rehabilitation units [3]. Patients participating in rehabilitation may experience falls, as they are being encouraged to be more independent and mobile and may over-stimulate their balance systems [4]. The topic of fall prevention has been emerging in recent literature on neurological patients [5, 6].

Fall prevention strategies, which rely on tailored multifactorial intervention programs, need to be based on the prior identification of patients at risk of falling, as reported in the systematic review from Cameron and colleagues [7]. Reliable tools for fall risk assessment would allow for actions on selected patients only, thus ensuring interventions to be both appropriate and cost effective. Various clinical tools for the identification of subjects at risk of falling have been published in the past two decades, mainly suited for use in geriatric wards, such as the Tinetti Performance Oriented Mobility Assessment, the Morse Fall Scale, the Berg Balance Scale, the St Thomas Risk Assessment Tool in Falling Elderly Inpatients (STRATIFY), the Conley scale and the Hendrich Fall Risk Model II (HIIFRM) [8–15]. Recently, there has been a spate of interest in falls risk assessment tools specific for stroke patients, such as the Stroke Assessment Fall Risk [5] and the 4-Item Falls Assessment Tool [6]. Nonetheless, no specific tools for a whole rehabilitation department have been provided. Among the above-mentioned tools, the HIIFRM is a multifactorial, eight-item tool that showed the best performance in terms of sensitivity and specificity. It has been validated by three independent studies on very large series in geriatric and acute care wards [14, 16, 17]. In addition, it can be carried out in just a few minutes [14, 18]. However, a description of its feasibility and predictive performance in rehabilitative patients is still missing in the literature.

The aim of the present paper is to address both the feasibility, i.e. the percentage of patients that can be assessed, and the predictive performance of the HIIFRM fall risk assessment tool in inpatient rehabilitation settings including orthopaedic, pulmonary and neurological rehabilitation wards.

## Methods

This prospective observational study was conducted during 6 consecutive months at the St. Sebastiano Hospital of Correggio – AUSL of Reggio Emilia, Italy. All adult inpatients admitted to the Orthopaedic Rehabilitation (OR), Pulmonary Rehabilitation (PR), Neurological Rehabilitation (NR) units were consecutively included in this study, without any exclusion criteria, in line with the aim of assessing the HIIFRM feasibility at admission.

All patients or relatives gave informed consent to data treatment in this research study and permission to publish anonymous data and results. The performance of this prospective study did not affect patients’ treatment in any way.

Patients’ risk of falling was evaluated by means of the HIIFRM tool within 24 h upon admission by two trained physiotherapists. The HIIFRM consists of eight weighted items assessing confusion/disorientation/impulsivity (score 4), symptomatic depression (score 2), altered elimination (score 1), dizziness or vertigo (score 1), male sex (score 1), antiepileptic prescription (score 2), benzodiazepine prescription (score 1), and “get up from chair” test (score ranging between 0 and 4). In this scale, the term altered elimination is qualified by the presence of any of the following symptoms: urinary or fecal incontinence, urgency or stress incontinence, diarrhea, frequent urination, and nocturia. The specific scores are based on their likelihood to cause a fall [14, 19]. These are summed up to a total score that can range between 0 (lowest risk) and 16 (highest risk). A patient is considered at high risk of falling if the total score is ≥5, which is the cutoff developed for geriatric patients [19]. According to the author, when the chair test cannot be administered due to the patient’s situation (e.g. recent hip surgery), the item associated is scored 0 [19]. Patients who cannot attempt the rising-from-chair test are classified as at-risk in the case of a total score from the remaining items equal to, or greater than the cutoff score.

The time used to administer the HIIFRM was recorded on a three level scale: ≤ 5 min, > 5 and ≤10 min, > 10 and ≤15 min.

The occurrence of falls was checked and recorded on a daily basis by professionals (nurses, physiotherapists, physicians), from their admission until discharge, death or transfer to another unit. According to the literature, a fall was registered when “an event which results in a person coming to rest inadvertently on the ground or floor or other lower level” took place [20].

### Data analysis

Descriptive statistics were used to assess the risk fall assessment feasibility in the sample as a whole and split by wards. The dependency of HIIFRM feasibility on age and length of the observation period was investigated by the non-parametric Mann-Whitney U-test. Its dependency on gender was assessed with the Chi-Square test (or the Fisher’s exact test as appropriate). The frequency of each HIFRM risk factor in the sample has been investigated and discussed.

The overall predictive power of the tool was obtained as the area under the ROC curve (AUC). A two-way table was fulfilled by entering subjects classified at risk (yes/no) according to the HIIFMR tool with the cutoff score of 5 by rows and subjects who experienced at least one fall in the hospital (yes/no) by columns. Sensitivity (Se) and specificity (Sp) of the scale were computed along with their 95% confidence intervals (95% CI), positive and negative predicted values (PPV, NPV). Next, the threshold that provides the best predictive power in our rehabilitative sample was found by applying the Hendrich’s classification rule (total score of the available items equal to or greater than the selected cutoff score) for all possible cutoffs. This procedure allowed considering the whole sample, including those patients unable to perform the rise-from-chair test. Finally, the predictive performances in terms of Se e Sp, PPV and NPV were assessed using the best cutoff found.

## Results

### Sample characteristics

There was a total number of 191 patients admitted to the selected wards and enrolled in this study during the 6 months of recruitment. Sample characteristics are reported in Table 1. These are presented for the sample as a whole and split by unit and HIIFRM assessment feasibility. As expected, the duration of the observation period was greater in NR, where more compromised patients were admitted.

**Table 1:** Sample characteristics at admission

	Sample Characteristic	Hendrich Fall Risk Model II at admission		
		Feasible	Not Feasible	Statistical comparison
*Admitted patients, n (%)*	191	147 (77%)	44 (23%)	
Ward NR/OR/PR, n	92/71/28	57/69/21	35/2/7	
Age, years, mean (SD)	66 (17)	69 (16)	58 (17)	*t = 3.97, p = 0.0001*
Gender, n (% female)	108 (57%)	86 (59%)	22 (50%)	
Observation period, mean days (SD)	36 ± 37	29 ± 30	68 ± 51	*t = −3.85, p = 0.0006*
Observed falls, n (%)	13 (7%)	11 (85%)	2 (15%)	
*Patients at NR Ward, n (%)*	92	57 (62%)	35 (38%)	
Stroke/TBI/Other, n	47/18/27	13/11/15	16/7/12	
Age, years, mean (SD)	59 (19)	62 (20)	54 (16)	*t = 2.15, p = 0.0348*
Gender, n (% female)	41 (45%)	27 (47%)	14 (40%)	
Observation period, mean days (SD)	60 (46)	49 (40)	86 (48)	*t = −2.92, p = 0.0070*
Observed falls n (%)	11 (12%)	9 (82%)	2 (18%)	
*Patients at OR Ward, n (%)*	71	69 (97%)	2 (3%)	
THR/TKR/Other, n	51/11/9	51/11/8	1/0/1	
Age, years, mean(SD)	75 (12)	75 (12)	85 (2)	*t = −5.08, p = 0.0009*
Gender, n (% female)	53 (75%)	51 (74%)	2 (100%)	
Observation period, mean days (SD)	18 (9)	18 (9)	17 (7)	*t = 0.16, p = 0.8951*
Observed falls, n (%)	2 (3%)	2 (100%)	0 (0%)	
*Patients at PR Ward, n (%)*	28	21 (75%)	7 (25%)	
RF/COPD/Other, n	11/7/10	7/7/7	4/0/3	
Age, years, mean(SD)	68 (9)	68 (9)	69 (12)	*t = −0.30, p = 0.7697*
Gender, n (% female)	14 (50%)	8 (38)	6 (86)	
Observation period, mean days (SD)	26 (29)	23 (27)	34 (35)	*t = −0.74, p = 0.4765*
Observed falls, n (%)	0 (0%)	0 (0%)	0 (0%)	

*NR* Neurological Rehabilitation, *OR* Orthopaedic Rehabilitation, *PR* Pulmonary Rehabilitation, *TBI* Traumatic Brain Injury, *THR* Total Hip Replacement, *TKR* total knee replacement, *RF* Respiratory Failure, *COPD* Chronic Obstructive Pulmonary Disease. The *t*-test was used for statistical comparisons

### Feasibility

Assessors succeeded in administering the HIIFRM scale in 147 cases of 191 (77%), as reported in Table 1. No adverse event took place during the administration of the scale. Failures in administering the HIIFRM were mainly related to minimally conscious states and vegetative states of patients with traumatic brain injury (TBI) (22 subjects). Remaining failures were related to the patient clinical situation (*n* = 17) (e.g.: bedridden patients with artificial nutrition) to psychiatric problems (*n* = 2) and to linguistic barriers (*n* = 2). Lastly, 1 patient refused participating in the study.

The majority of failures (35/44, 80%) took place at the NR unit in subjects who had a longer observation period (*p* = 0.007). These were younger than the rest of the neurological sample (*p* = 0.034) and were mainly subject with TBI consequent to road accident. Feasibility approached 100% at OR, but was limited to 75% at PR.

The administration time resulted comprised between 5 and 10 min in the majority of cases. The most time-consuming items were, as expected, the rise-from-chair test and the analysis of pharmacologic treatments.

### Fall history

Out of the 147 screened patients, 11 fell during hospitalizion (7.5%), 7 males and 4 females. Mean age (SD) was 63 (22) years (range 20–87) and mean observation time was 52 (23) days. Nine falls occurred to patients of the NR and the remaining two to patients of the OR, while no falls occurred in the PR unit (Table 1). The fall rate was 3.43/1000 patient days at NR and 1.62/1000 patient days at OR. Among fallers at NR, 3 were young adults, with ages of 20, 21 and 41 years. It appears from our data that falls in a rehabilitative hospital mainly take place among neurological patients and may involve young subjects, too.

### HIIFRM risk factors occurrence and total score distribution

The occurrence of single risk factors assessed by the HIIFRM is presented in Table 2, which indirectly shows the difference in the case-mix among wards. The scores (weights) corresponding to each item in the HIIFRM tool are also reported.

**Table 2:** Frequency of each HIIFRM fall risk item in the three units

Item	Item Score	Occurrence (%)		
		NR	OR	PR
Confusion /Disorder/Impulsivity	(4)	40	19	10
Symptomatic Depression	(2)	23	28	24
Altered Elimination	(1)	28	49	57
Dizziness/Vertigo	(1)	84	46	48
Male Gender	(1)	53	26	62
Antiepileptics	(2)	18	9	14
Benzodiazepines	(1)	44	26	52
chair test = 0	(0)	7	3	47
chair test = 1	(1)	7	4	29
chair test = 3	(3)	5	3	5
chair test = 4	(4)	76	22	14
chair test = not feasible	(0)	5	66	5

*NR* Neurological Rehabilitation (*N* = 57), *OR* Orthopaedic Rehabilitation (*N* = 69), *PR* Pulmonary Rehabilitation (*N* = 21)

The large majority of neurological patients needed for assistance to stand up from a chair (score 4), and suffered from dizziness or vertigo, which scores 1. Moreover, about 50% of patients were male (score 1) and 40% had a confusion or disorientation or impulsivity (score 4). As a consequence, a very large number of NR subjects exceeded the threshold level of 5 and was classified as at risk of falling. Conversely, high score items were not frequent in patients at OR and PR wards. Hence, the contemporary presence of many low-score risk factors was required to reach the threshold score.

The chair test was not feasible in most of the orthopaedic patients, whose most frequent risk factors were altered elimination and dizziness or vertigo. In patients at pulmonary rehabilitation male sex, altered elimination use of benzodiazepines and dizziness or vertigo had a frequency ranging between about 50 and 60%.

The rise-from-chair test was relatively easy for patients at PR, while it was frequently impossible to be administered at the OR ward, mainly due to recent surgery, such as total hip or knee replacements. The presence of depression and the use of antiepileptic drugs were similar among wards, altered elimination at OR and PR was about twice frequent than at NR and the use of benzodiazepines was lower at OR compared to the other two wards.

**Fig. 1:**
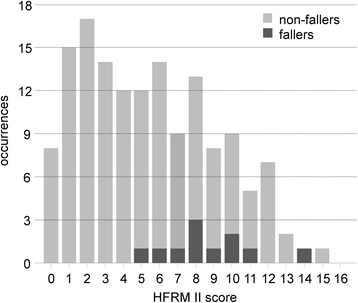
HIIFRM score distribution in the sample (*N* = 147) for both fallers (*N* = 11) and non-fallers

Finally, the HIIFRM total score distribution is reported in the histogram of Fig. 1. HIIFRM score ranged between 0 and 15 in non-fallen subjects and between 5 and 14 in those who fell. It is evident in Fig. 1 the overlap of scores obtained by fallers and non-fallers. The median score was 8 for fallers and 5 for non-fallers (*p* = 0.0021, Mann-Whitney U test).

### HIIFRM predictive performance

**Fig. 2:**
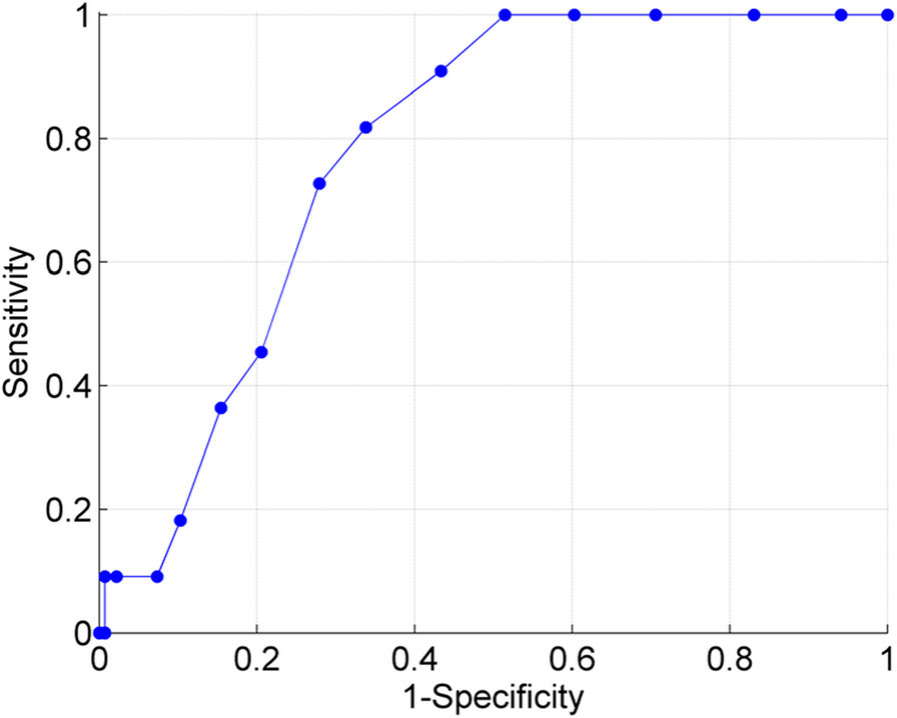
Receiver Operating Characteristic (ROC) Curve

Predictive performance was computed based on 11 falls from 147 subjects. The ROC curve is reported in Fig. 2. The area under the curve was AUC = 0.779, *p* = 0.002*,* (95% CI: 0.685−0.873), thus indicating a moderate predictive power of the scale.

Based on the standard HIIFRM classification procedure (score≥5 means at risk for falling) all the 11 fallers were properly categorized, leading to a sensitivity of 100% with a specificity as low as 49%, as reported in Table 3.

**Table 3:** Predictive ability of the HIIFRM administered at the investigated rehabilitative setting

		Observed Falls		
		**+**	**–**	Tot.
Predicted Falls	+	11	70	81
	**–**	0	66	66
	Tot.	11	136	147
			95% C.I.	
Se (%)		100	N/A	N/A
Sp (%)		49	40	57
PPV (%)		14	6	21
NPV (%)		100	N/A	N/A

*Se* Sensitivity, *Sp* Specificity, *PPV, NPV* Positive and Negative Predicted Values, *C.I.* Confidence Interval, *N/A* Not Applicable

The effect of higher cutoff scores on the classification along with the consequent sensitivity and specificity is presented in the two-way tables reported in Table 4. As expected, sensitivity progressively decreased and specificity progressively increased when the cutoff score increased.

**Table 4:** Predictive ability of the HIIFRM at subsequent cutoff scores from 6 to 9

		Cutoff = 6			Cutoff = 7			Cutoff = 8			Cutoff = 9		
		Observed Falls			Observed Falls			Observed Falls			Observed Falls		
		**+**	**–**	tot	**+**	**–**	tot	**+**	**–**	tot	**+**	**–**	tot
Predicted Falls	+	10	59	69	9	46	55	8	38	46	5	28	33
	–	1	77	78	2	90	92	3	98	101	6	108	114
	tot	11	136	147	11	136	147	11	136	147	11	136	147
			C.I. 95%			C.I. 95%			C.I. 95%			C.I. 95%	
Se (%)		91	74	100	82	59	100	73	46	99	45	16	75
Sp (%)		57	48	65	66	58	74	72	65	80	79	73	86
PPV (%)		14	6	23	16	7	26	17	6	28	15	3	27
NPV (%)		99	96	100	98	95	100	97	94	100	95	91	99

*Se* Sensitivity, *Sp* Specificity, *PPV*, *NPV* Positive and Negative Predicted Values, *C.I.* Confidence Interval

The best trade-off was obtained by applying a cutoff of 8, that is by considering at risk for fall subjects with a total score ≥ 8 and subjects who were not able to perform the get up from chair test with a sum of the other items ≥8. By using this cutoff, a sensitivity of 73% and a specificity of 72% were obtained, which are similar to those obtained in the acute care settings by using the original HIIFRM cutoff [14]. A cutoff of 7 would provide a better sensitivity 82% at the cost of a lower specificity 66% that is of a greater number of patients to be included into the fall prevention programs. Conversely, a cutoff equal to 9 would dramatically reduce sensitivity, thus being not adequate.

The two subject who fell at the OR ward had a total score of 7 and 11, respectively. Therefore, they would be properly classified by using the cutoff score of 7.

## Discussion

This study aimed at addressing feasibility and predictive performance of the HIIFRM, when used in a rehabilitative department including units of different specialization. The main result is that the HIIFRM was satisfactory in terms of both feasibility (around 80%) and predictive power (AUC = 0.779) when used at rehabilitative neurologic, orthopaedic and pulmonary units.

In our sample the 7.5% of the admitted patients fell. This value is similar to that reported for high-risk non-geriatric medical wards [21]. This confirms the need for screening procedures and prevention strategies at rehabilitative wards. The HIIFRM was selected in this study because of both its multifactorial structure and the satisfactory predictive performance in the assessment of inpatients in medical, surgical and geriatric wards, which has been outlined by several independent studies on wide samples and by recent systematic revisions [14, 16, 18, 22–24]. Along with the inpatient rehabilitative wards, these are all the hospital wards where it is reasonable to seek to identify patients at risk of falling by a tool, as this event is not rare. The possibility of extending the use of HIIFRM to the rehabilitative settings too, would allow utilizing a unique fall risk assessment tool across all wards with high fall occurrence. This would be easy to implement in hospitals and should enhance compliance of nurses and other professionals [6].

In our study, HIIFRM was successfully administered to nearly the 80% of patients admitted to the NR, OR and PR wards during the six months covered by the study, and the administration time was usually between 5 and 10 min. We consider this result satisfactory. The majority of failures in tool administration took place in the NR unit and was related to minimally conscious patients and vegetative states at admission. These patients are not at risk of falling as they cannot leave the bed and would not be included in a screening procedure at least until volitional movements reappear. The presence of highly compromised patients (e.g. with respiratory failure) limited the feasibility at PR (75%). Feasibility approached 100% at the OR ward. Thanks to the HIIFRM classifying procedure, the fall risk status can be assessed also for patients who underwent orthopaedic surgery before admission, such as a total hip arthroplasty, despite of their inability in performing the functional task. As recommended, all unavailable patients (of NR and OR wards) have to be reassessed as soon as safely permitted by their clinical condition [14]. Feasibility was not investigated by previous studies on fall risk assessment tools, hence a comparison with literature cannot be carried out.

The “get up from chair” test deserves further considerations. It was not included in the first version of the Hendrich Fall Risk Tool [25] and has been introduced in the HIIFRM to increase the predictive power of the tool [14]. It indirectly measures the residual ability of generating force in the lower limbs. This ability has been progressively recognized as a requisite for counteracting the unexpected imbalance that may lead to falls [26] and its evaluation boosts the predictive performance of clinical tools for fall-risk assessments [27–29].

The predictive power of the HIIFRM found is this study was moderate, with AUC approaching 0.8 and similar to those obtained in studies on both general [6, 17] and older acute care settings [18, 29]. Interestingly, the performance of the HIIFRM resulted similar to that obtained by patient-specific tools assessed by the recent literature [5, 6, 30, 31]. Along with predictive power, the identification of a tool with good specificity is of major importance to plan both screening procedures and sustainable prevention pathways. According to our results (Table 4), the relationship between “real fallen” subjects and “expected fallen” subject is about 1 to 5 when used for cutoffs of 7 and 8, which is similar to what has been obtained in the geriatric field [18] but worse than those obtained in neurologic field with SAFR scale [5].

The results of the present study show that thresholds of 7 and 8 provide the best compromise between sensitivity and specificity in the investigated settings (See Table 4). By using the cutoff of 7 (i.e. by considering at risk a patient with a total score ≥ 7), the performance of the HIIFRM resulted satisfactory, with sensitivity superior than 80% and specificity approaching 70%. The predictive capacity is better than the one obtained with the SAFR scale, specific for patients with stroke (*N* = 446, Se = 78%, Sp = 63%) [5]. The selection for a cutoff value between 7 and 8 should be made according to the available hospital resources. An increase in threshold reduces false positives and therefore makes it less burdensome and more feasible the implementation in prevention protocols for those identified at risk.

In the NR ward, the items dealing with pathologies of the central nervous system were frequent (see Table 2), while items typical for older patients were less frequent, such as incontinence and depression. In this ward, the two items that score 4, that is confusion and the inability to rise from chair without assistance, were found in many patients. As a consequence, the average score at NR was particularly high and the majority of NR patients presented a total score greater than 5, thus resulting at high risk of falling according to the HIIFRM classification rule. This explains the 100% sensitivity obtained with the cutoff of 5, along with the scarce specificity, and the need for higher cutoffs (See Table 3).

The proportion of patients who fell was as high as 18% at the NR ward, well in accordance with the values (14−25%) reported in the literature for similar patients [5, 22, 30]. Interestingly, fallers at NR were younger than non-fallers (See Table 1). The well-known relationship between older age and fall-risk may not extend to the whole inpatient rehabilitation population. Younger people could reasonably be more active or more prone to attempt ‘risky’ behaviours, such as unassisted standing or walking, as reported by Breisinger and colleagues, who analysed inpatient stroke patients and found similar results [5]. The higher fall occurrence at NR might be also accounted for by the longer stay and observation period of neurological patients (see Table 1).

A lower occurrence of falls - 3% - has been registered at the OR ward, again in line with data available from the literature, where fall occurrences between 2% and 17% have been described [32]. No falls occurred at the PR ward, in our study. This result could be explained by the case-mix, where patients were either highly compromised and bedridden or with good functional ability. This can be confirmed by observing the variability in the length of stay of these patients reported in Table 1. In the OR ward, the most frequent items were the inability in performing the chair test, altered elimination and dizziness, in line with both age and type of admitted patients (see Table 1). The occurrence of risk factors at OR was similar to that reported by Ivziku for a sample of geriatric patients [18]. In the PR ward, patients mainly presented altered elimination and dizziness/vertigo, while the occurrence of the items assessing confusion and lower limb weakness was very low. Thus, the HIIFRM risk factors with the greatest score were rare and the total score for these patients was in general low.

Finally, whilst a pathology-specific tool could be appropriate where a very narrow case-mix of patients is admitted, such as in a stroke unit, a multifactorial tool is to be preferred where a wide case-mix of patients is treated, as in the case of a rehabilitation department.

### Limitations of the study

The main limitation of this study is the low number of subjects included and of falls recorded. This would suggest caution in generalizing our results, even if the fall rate was congruent to those in the literature. A further weakness is that patients were assessed at admission only, according to the aim of the study. Hence, eventual changes in the clinical conditions and in the consequent fall risk status have not been considered.

## Conclusions

In conclusion, the HIIFRM showed satisfactory feasibility and predictive performances in the assessment of fall-risk in rehabilitative settings. Hence, apart from being used in geriatric, in the long-term care, medicine, and surgery departments with a cutoff of 5, the HIIFRM could also be used to determine the risk of falling of hospitalized patients in rehabilitation departments (i.e. orthopaedics, pulmonary and neurology) adopting a specific cutoff. Based on both available literature on geriatric patients and our findings in the rehabilitative wards, we propose the assessment of fall risk amongst all hospital units with high fall occurrence by means of a unique tool, the HIIFRM, with two different cutoff values.

## Abbreviations

AUC: Area Under the Curve
HIIFRM: Hendrich Fall Risk Model II
NPV: Negative Predictive Value
NR: Neurologic Rehabilitation
OR: Orthopaedic Rehabilitation
PPV: Positive Predictive value
PR: Pulmonary Rehabilitation
ROC: Receiver Operating Characteristic
Se: Sensitivity
Sp: Specificity
STRATIFY: St Thomas Risk Assessment Tool in Falling Elderly Inpatients
